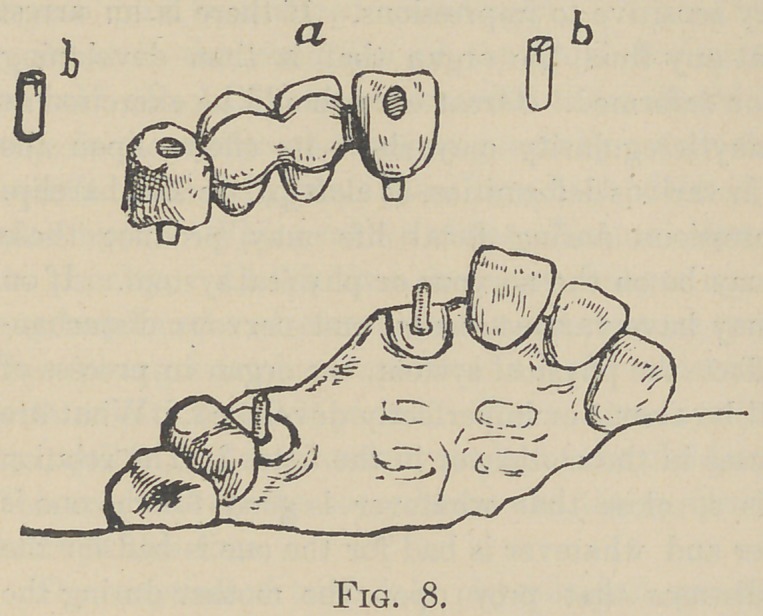# Crown and Bridge-Work

**Published:** 1888-07

**Authors:** C. S. Case

**Affiliations:** Jackson, Mich.


### Crown and Bridge-Work.

BY C. S. CASE M.D., D.D.S., JACKSON, MICH.

(Read before the Michigan State Dental Association at Ann Arbor, Mich., March 20, 1888.)

Fig. 1.

Fig. 2.

I wish to tell you about a new crown and movable bridge denture. The crown proper was described in a paper read before the Chicago Dental Infirmary clinic in December, and appeared in the January number of the *Dental Review*. Those who are doing porcelain work can make the entire portions. It is hoped, however, to have all the different parts in the market ready for use.

Select a plate tooth with pins standing side by side. Bend these around a platinum tube of the proper size as in Fig. 1, and restore the lingual contour with porcelain. You will now have a crown with a tube passing vertically through its centre similar to the Ash & Son's pivot tooth.

Before describing the operation of setting the crown, let me first call your attention to Fig. 2, which represents a vertical section of the root and crown displaced. *E* is a screw-threaded post seated in the root *R*. *A* is the porcelain tooth crown possessing the tube *B*. *C* is a second tube —preferably of platinum—which is soldered or screwed into tube *B*, forming a shoulder therein, and, projecting from the tooth, is finally received into the annular cavity, *r*, in the root,—the latter device being intended to give a longer and more rigid bearing to the crown. *D* is another tube threaded internally which, when screwed on to the post, will pass into the tube *B*, and being received against the shoulder, formed by the tube *C*, the parts can be firmly drawn to position as in Fig. 3, which represents the projecting ends of the post and tube cut away and the surface polished even with the lingual surface of the crown.

Fig. 3.

It is presumed that the operator will be supplied with drills, taps, posts and tubing of such exact size that the parts will work together with mechanical percision.

Fig. 4.

The tubes can be made by cutting narrow strips two or three inches long, from platinum plate of the proper thickness, and passing them through a draw-plate,—remembering that the internal diameter of tube *D* must be equal to the smallest diameter of the post, (to allow for the thread,) that that of tube *C* sufficient to slide over the post, and the external diameters of these two tubes equal, and a trifle less, than the internal diameter of tube *B* in the crown. Finish the tubes by soldering the edges which have been drawn together with a minimum quantity of pure gold. If the How post is used the tap which is used for the root will answer to cut the thread for the nut, or tube *D*. This should be cut only sufficient for each time of using.

Fig. 5.

Thus far I have used the A How post, though my original design calls for a post having a cone-shaped screw at one end very coarsely threaded for the root, the base of the cone being equal in diameter to the requirements of the annular cavity, *r*, Fig. 4; the projecting portion of the post, straight and finely threaded to engage with the nut. This by its minimum diameter will afford the greatest possible strength in proportion to its size, and possess threads in keeping with the requirements of the material with which they are intended to engage.

The first step in the operation proper, is to dress off the end of the root so that its exposed surface shall be a little below, but parallel with, the festooned border of the gum, (Fig. 5,) and to fit the selected or prepared crown to it so that, when in position, there will be no rocking movement. While holding the crown in place pass a drill or other suitable instrument through the tube, and mark on the end of the root the starting place to drill for the post. If the root canal is not in the proper position to receive the post, or is enlarged by decay or otherwise, it should first be filled with gold or amalgam.

Drill and tap for post and ream for annular cavity, *r*. After the post has been fixed in the root it may be necessary to bend it slightly in order that it may pass through the centre of the tube *B*. Now, to finally prepare the crown, insert tube *C*, a short distance into the cervical end of tube *B*, and invest,—leaving exposed the part where the tube enters. Use a minimum quantity of some easily flowing hard solder and apply the heat from the under side until it disappears between the tubes. I have used soft solder, but the use of the other is very easy and more reliable. Again, if tube *C* is made of plate of the same thickness as tube *D*, its external diameter will be larger, and can be threaded to screw into the previously tapped crown, tube *B*, instead of soldering. Cut off the tube *C* the proper length for the cavity *r*, and fit the parts together by screwing on the coupling, tube *D*, which you will have prepared as directed.

Before the final adjustment apply gutta-percha or Hill's stopping to the end of the root or crown; thoroughly warm the latter and immediately bring the parts firmly together.

The reason the ordinary cheap crown fails and early becomes a disgusting thing to both patient and operator, is because of decomposing organic material, that almost invariably will work its way between the joints with ultimate decay of the root. This is mainly due to the large mass of imbedding material which they require,—usually amalgam that shrinks or is not held firmly in place until it hardens, together with the general instability of the fittings, which admit of a slight movement during mastication with final imperfection of the joint.

If it is desired to surround the root with a band in connection with this crown it can be accomplished with comparative ease in several ways. It can be attached to the crown as in Fig. 6. *F* is the band or ferrule that has been fitted to the root and the crown ground to fit into it. *P* is thin platinum plate which is soldered to tube *C* and the band *F*.

Fig. 6.

Again, the band may be provided with lugs which will prevent it from being forced too far on to the root, then the crown can be ground and drawn firmly into it with the final coupling.

There is another way presented for using the band especially applicable where this method is used for bridge-work, which is as perfect for protecting the root and possesses the advantage, also, of not having it in sight.

Dress the root the same as at first described and make band of very thin platinum plate. While on trim off the projecting edge even with the dressed face of the root. Bend or burnish a piece of platinum plate (also very thin) against the end of the root, its edges extending beyond the band. Remove band, solder the two together and finish. Cover the inside of this cap with a layer of chloro-percha, return to place and burnish tightly against the sides of the root. Now fit the crown as at first.

Fig. 7.

BRIDGE-WORK.

For partial bridge dentures between the roots of anterior teeth where the bite is not short, the following method, in connection with this crown will be found of great value, especially as it admits of being removed for repairs or uncorking a root.

After selecting and preparing the fronts for the supporting crowns as in Fig. 1, solder the end of a narrow strip of platinum plate to the tube so that when restored with porcelain, its end will project from the crown on the bridge side—or from both sides if desirable, as in Fig. 7—and uniting with its fellow from the opposite crown will form a span to which the intervening plate teeth may be rivited and soldered. In Fig. 8, *a* represents a partial bridge denture, and *b b*, the coupling nuts. The latter can be made from the coupling tube, *D*, by first tapping it and cutting off pieces the proper length, which are made solid at one end and cut so as to be worked with a screwdriver. On account of the force required to unscrew them, the heads of the nuts should be of iridio-platinum or some hard metal.

## Figures and Tables

**Fig. 1. f1:**
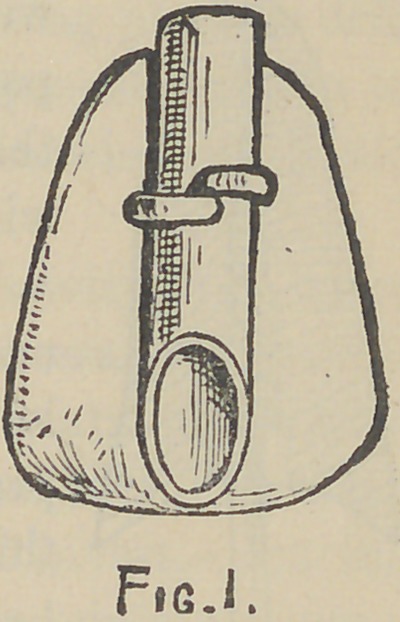


**Fig. 2. f2:**
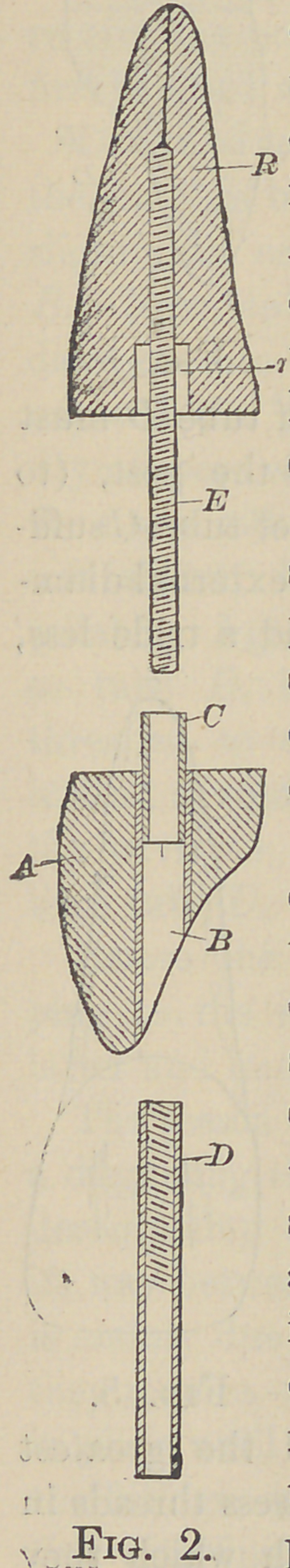


**Fig. 3. f3:**
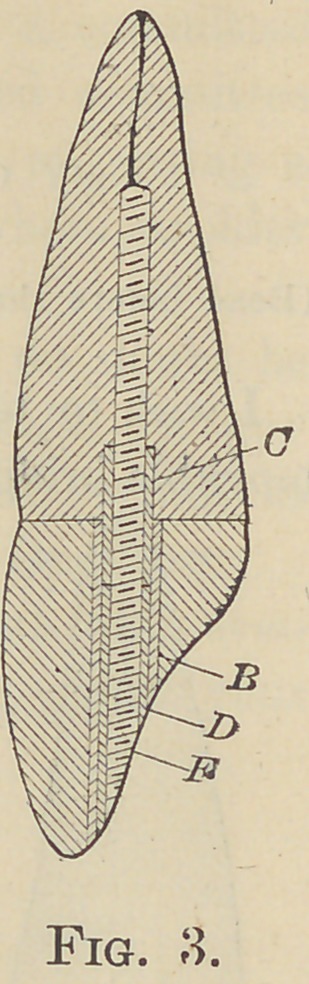


**Fig. 4. f4:**
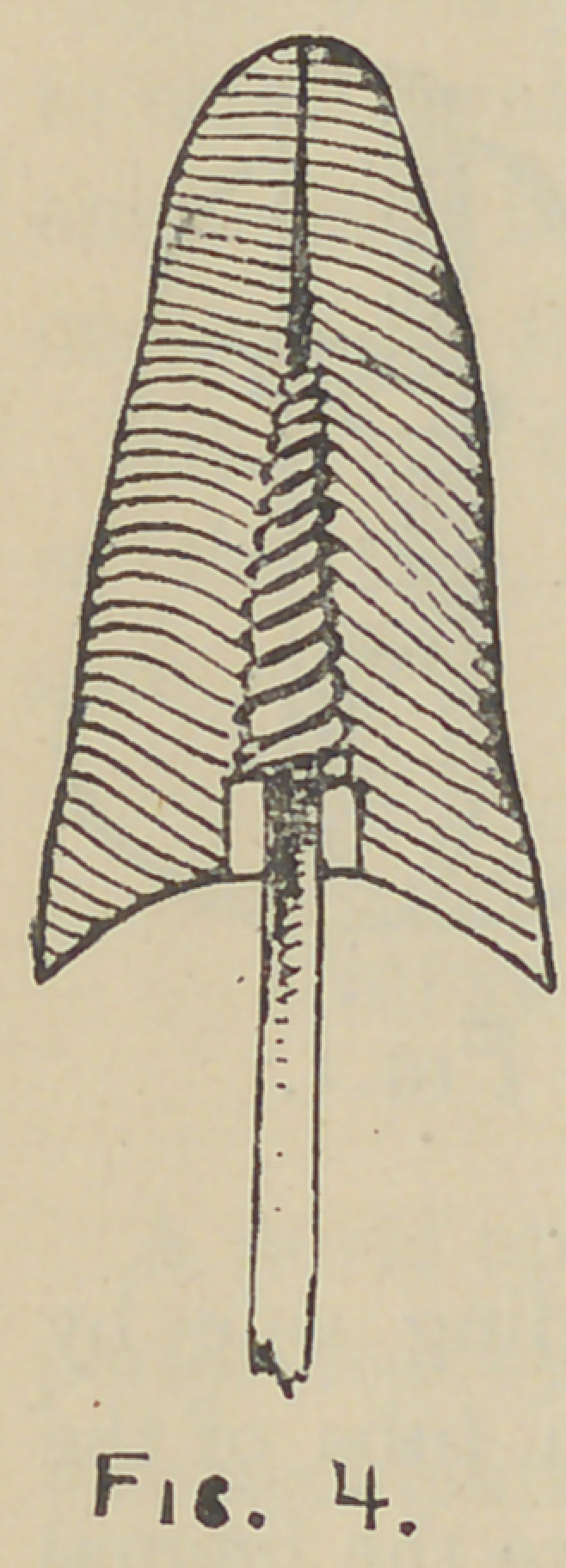


**Fig. 5. f5:**
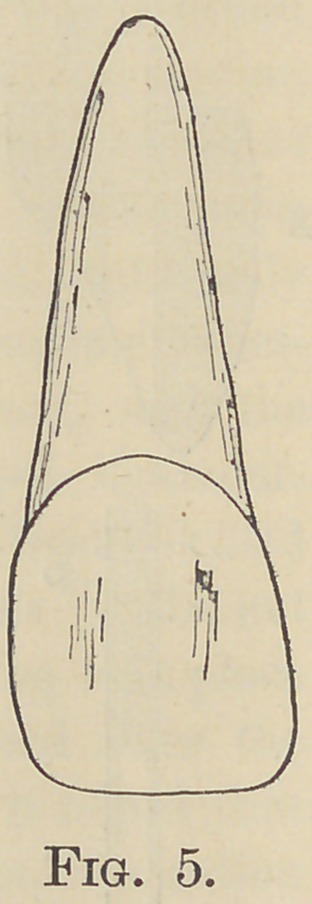


**Fig. 6. f6:**
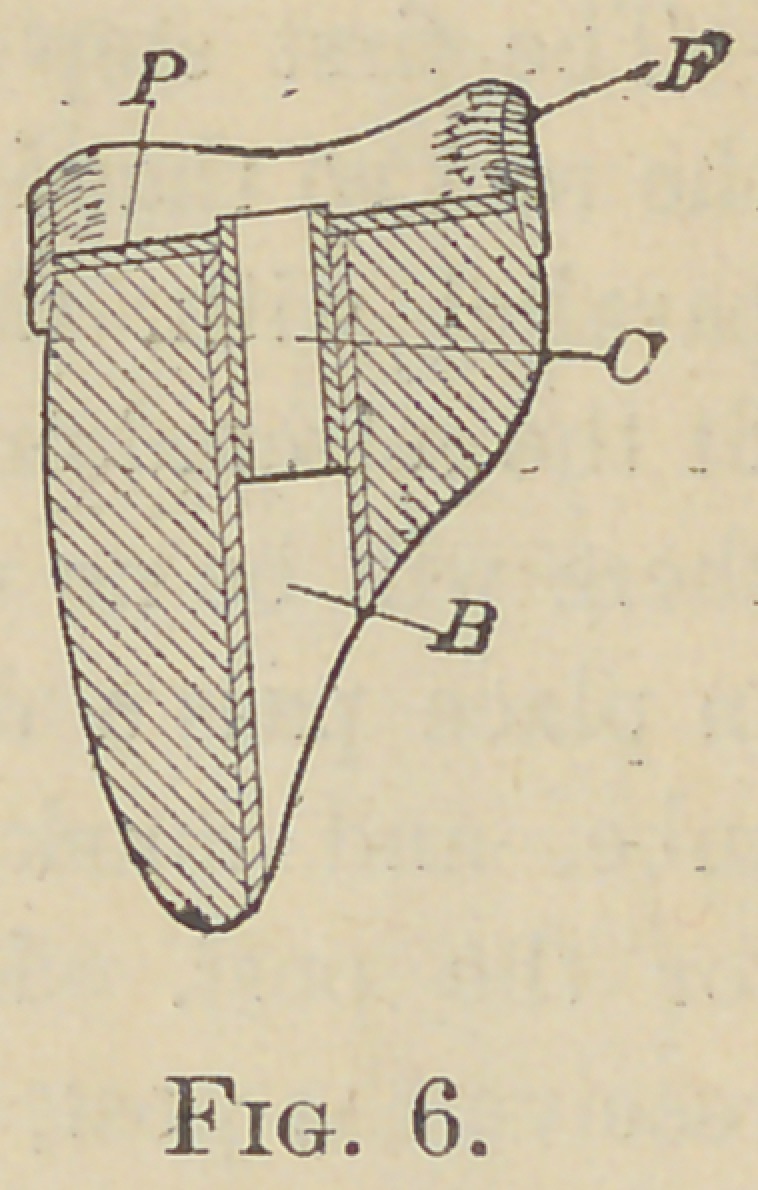


**Fig. 7. f7:**
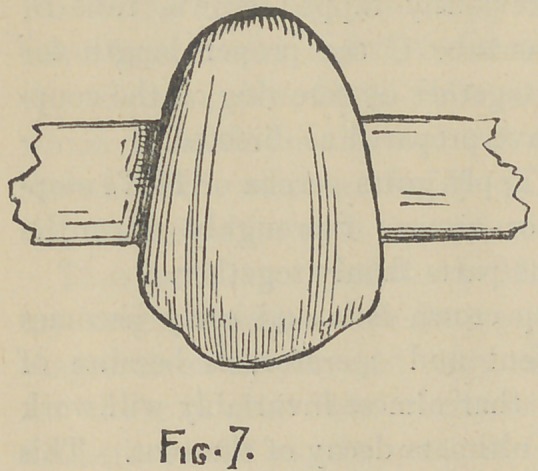


**Fig. 8. f8:**